# Traumatic perilymphatic fistula with stapes luxation into the vestibule and pneumolabyrinth: a case report

**DOI:** 10.1093/jscr/rjag072

**Published:** 2026-07-11

**Authors:** Omar Oulghoul, Salah-eddine Youbi, Youssef Lakhdar, Mohammed Chehbouni, Othmane Benhoummad, Youssef Rochdi, Abdelaziz Raji

**Affiliations:** Department of ENT Head and Neck Surgery, Faculty of Medicine and Pharmacy of Marrakech, Cadi Ayyad University, University Hospital Center Mohammed VI, Marrakech, 40 000, Morocco; Department of ENT Head and Neck Surgery, Faculty of Medicine and Pharmacy of Marrakech, Cadi Ayyad University, University Hospital Center Mohammed VI, Marrakech, 40 000, Morocco; Department of ENT Head and Neck Surgery, Faculty of Medicine and Pharmacy of Marrakech, Cadi Ayyad University, University Hospital Center Mohammed VI, Marrakech, 40 000, Morocco; Department of ENT Head and Neck Surgery, Faculty of Medicine and Pharmacy of Marrakech, Cadi Ayyad University, University Hospital Center Mohammed VI, Marrakech, 40 000, Morocco; Department of ENT Head and Neck Surgery, Faculty of Medicine and Pharmacy of Agadir, Ibn Zohr University, University Hospital Center Souss Massa Agadir, 80 000, Morocco; Department of ENT Head and Neck Surgery, Faculty of Medicine and Pharmacy of Marrakech, Cadi Ayyad University, University Hospital Center Mohammed VI, Marrakech, 40 000, Morocco; Department of ENT Head and Neck Surgery, Faculty of Medicine and Pharmacy of Marrakech, Cadi Ayyad University, University Hospital Center Mohammed VI, Marrakech, 40 000, Morocco

**Keywords:** ear trauma, perilymphatic fistula, stapedovestibular dislocation, pneumolabyrinth, stapes surgery

## Abstract

Traumatic perilymphatic fistula with disruption of the annular ligament and stapedovestibular dislocation is extremely rare. Leakage of perilymph can cause inner ear damage and dysfunction. Treatment remains controversial due to the limited number of reported cases in the literature. Here we report the case of a penetrating ear trauma causing a perilymphatic fistula with stapedovestibular dislocation, stapes displacement into the vestibule and pneumolabyrinth. We discuss clinical aspects, imaging features, management, and functional outcomes.

## Introduction

Perilymphatic fistula (PLF) is an abnormal communication between the inner ear and middle ear, secondary to a dehiscence in the otic capsule, oval, or round window, causing leakage of perilymph [1]. This can result in cochlear or vestibular symptoms, such as sensorineural hearing loss, tinnitus, vertigo, and imbalance. It can be categorized as either congenital, or acquired which includes iatrogenic (stapes surgery), traumatic (temporal bone fracture), erosive (cholesteatoma or middle ear malignancies), and idiopathic [1]. Traumatic perilymphatic fistula secondary to stapes dislocation from the oval window is extremely rare for two main reasons: first is the annular ligament that firmly attaches the stapes footplate to the oval window, and second is the deep location of the stapes in the tympanic cavity which makes it less vulnerable to traumatic injury [2].

Here we report the case of a penetrating ear trauma causing a perilymphatic fistula with stapedovestibular dislocation, stapes displacement into the vestibule and pneumolabyrinth. We discuss clinical aspects, imaging features, treatment options, and functional outcomes.

## Case report

A 38-year-old man, with no relevant medical history, presented to the Emergency Department with acute onset of vertigo, imbalance, otalgia, left-sided unilateral hearing loss, and non-pulsatile tinnitus, after a small tree branch penetrated his left external auditory meatus by accident. On examination, his vitals were stable, and there was no sign of major head or cervical spine trauma.

The patient presented a spontaneous right-beating nystagmus with a positive fistula test on the left side. Romberg’s test revealed instability and left deviation. Otoscopy showed a small perforation in the posterosuperior quadrant of the left tympanic membrane. The long process of the incus was visible through the perforation, and the incudo-stapedial joint appeared to be dislocated (Fig. 1). In addition, there was no sign of facial nerve paralysis.

**Figure 1:**
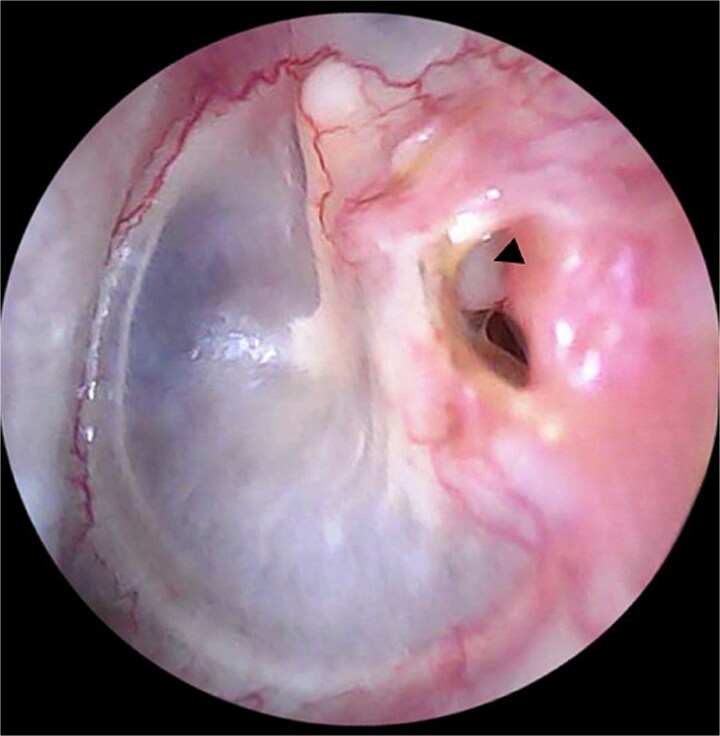
Otoscopic examination showing a small perforation in the posterosuperior quadrant of the left tympanic membrane. The long process of the incus (arrow head) is visible through the perforation.

The patient was then transferred to our Otolaryngology Head and Neck Surgery Department for further evaluation. Pure-tone audiometry revealed a moderately severe conductive hearing loss on the left side with an average threshold of 62.5 dB HL and air-bone gap of 41.6 dB HL (Fig. 2). Weber’s test showed a clear lateralization toward the left side.

**Figure 2:**
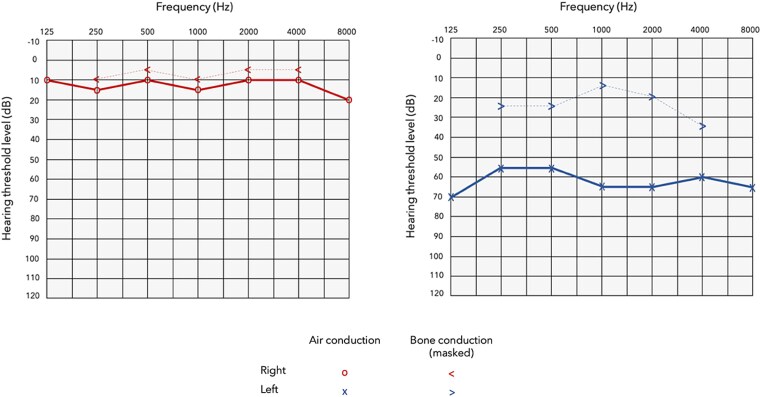
Audiogram demonstrating a moderately severe conductive hearing loss on the left side, and normal hearing on the right side.

High resolution computed tomography (HRCT) of the temporal bone demonstrated a disruption of the incudo-stapedial joint, with the long process of the incus directed superiorly, toward the tympanic segment of the facial nerve. The stapes footplate was dislocated from the oval window, and deeply depressed into the vestibule. In addition, we noted the presence of air bubbles inside the vestibule and horizontal semicircular canal, suggestive of pneumolabyrinth which is a sign of perilymphatic fistula (Figs 3 and 4).

**Figure 3:**
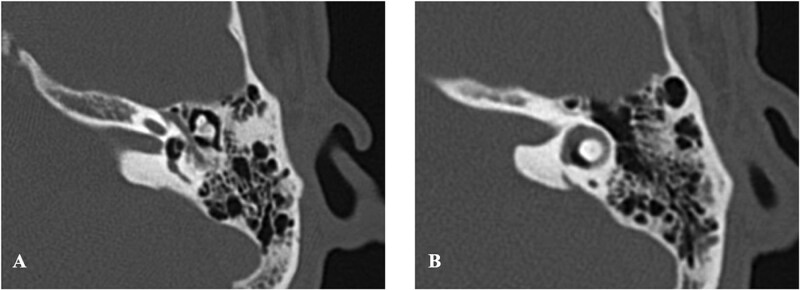
Axial HRCT of the left temporal bone showing medial displacement of the stapes into the vestibule (A), and the presence of air bubbles (pneumolabyrinth) inside the vestibule (A) and horizontal semicircular canal (B).

**Figure 4:**
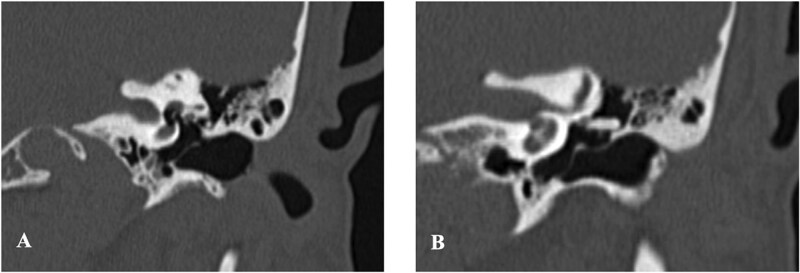
Coronal HRCT of the left temporal bone illustrating stapes footplate depression into the vestibule (A), and disruption of the incudostapedial joint with the long process of the incus directed superiorly toward the tympanic segment of the facial nerve (B).

The patient’s vestibular symptoms persisted after 5 days of symptomatic treatment (analgesics, vestibular suppressants, antibioprophylaxis, and corticosteroids). Consequently, he was scheduled for exploratory tympanotomy to seal the fistula and prevent potential spread of infection to the inner ear. Intraoperatively, after careful removal of the stapes from the vestibule (Fig. 5), we sealed the fistula at the level of the oval window with a temporalis fascia graft, then we placed a Teflon ® piston between the lenticular process of the incus and the sealed oval window, to maintain ossicular chain function. We also conducted a myringoplasty using a temporalis fascia graft to repair the perforation of the tympanic membrane.

**Figure 5:**
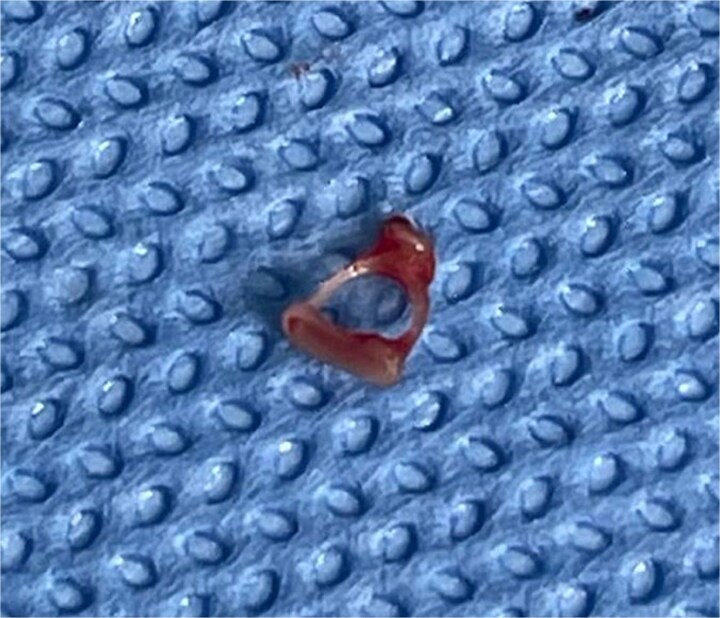
Intact stapes after careful removal from the vestibule.

No intraoperative incidents nor postoperative complications were noted and the patient’s symptoms started to resolve progressively after surgery. Postoperative visit 6 weeks after surgery revealed a completely healed tympanic membrane (Fig. 6) and audiogram indicated improvement in auditory function with a significant air-bone gap closure and no deterioration in bone conduction thresholds (Fig. 7).

**Figure 6:**
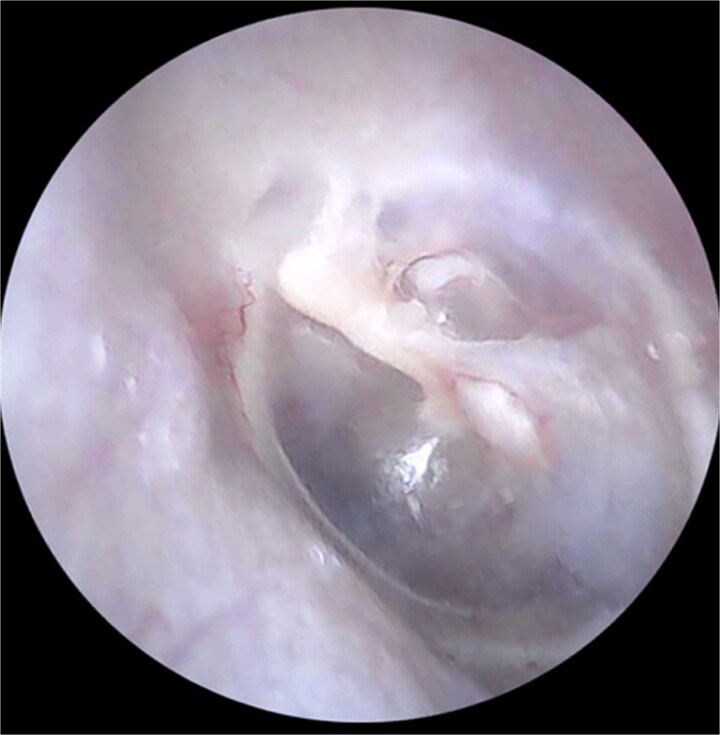
Otoscopic examination after surgery showing complete healing of the tympanic membrane perforation.

**Figure 7:**
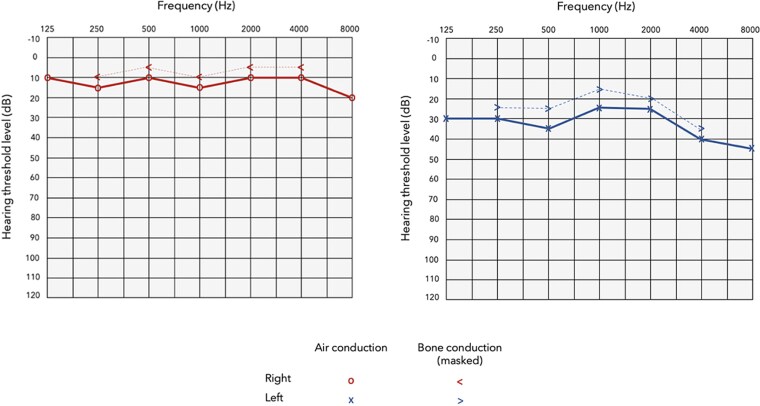
Postoperative audiogram indicating improvement in auditory function with significant air-bone gap closure and preservation of bone conduction thresholds.

Temporal bone HRCT obtained 6 months after surgery showed proper placement of the piston into the oval window and complete regression of the pneumolabyrinth (Fig. 8).

**Figure 8:**
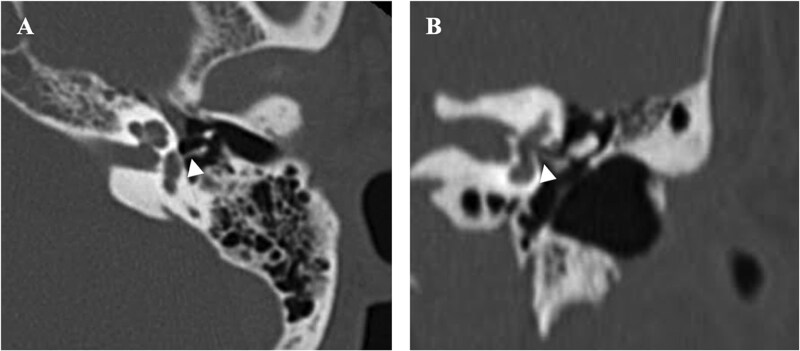
Postoperative HRCT obtained 6 months after surgery (A): Axial, (B): Coronal, indicating complete regression of the pneumolabyrinth and proper placement of the piston into the oval window (arrowhead).

## Discussion

Traumatic perilymphatic fistula (PLF) secondary to stapes dislocation from the oval window is extremely rare for two main reasons: first is the annular ligament that firmly attaches the stapes footplate to the oval window, and second is the deep location of the stapes in the tympanic cavity which makes it less vulnerable to traumatic injury [2].

Perilymph leakage from the fistula can result in cochlear and/or vestibular symptoms such as sensorineural hearing loss, tinnitus, vertigo, and imbalance.

The most characteristic sign on CT is pneumolabyrinth, which is defined by the presence of air bubbles inside the cochlea, vestibule, or semicircular canals [3].

Contrary to idiopathic fistula, traumatic annular ligament disruption may cause important perilymph leakage leading to inner ear damage and dysfunction [4]. There is no established treatment protocol due to the limited number of reported cases. Early surgical intervention is recommended for patients with persistent or progressive symptoms, to locate and seal the fistula and prevent further inner ear damage. On the other hand, for patients with stable hearing levels and spontaneous improvement of vestibular symptoms, conservative treatment with close monitoring of hearing function can yield good functional outcomes.

The condition of the stapes is a key factor for surgical decision: whether it is intact or fractured, and how deeply displaced it is inside the vestibule, determines treatment options, surgical approach, and outcome [5]. Some studies argue that stapes removal is not necessary since the process is associated with a risk of additional inner ear damage. On the contrary, other studies suggest that it is better to remove the stapes from the vestibule, before the onset of fibrosis that might cause vestibular obstruction and inner ear dysfunction [6].

In our case, we removed the stapes from the vestibule and sealed the fistula in the oval window with a temporalis fascia graft. We placed a Teflon® piston between the sealed oval window and the lenticular process of the incus to maintain ossicular chain function, then we repaired the tympanic membrane perforation with a temporalis fascia graft. After surgery, the outcome was complete resolution of vestibular symptoms with preservation of inner ear function.

Uehara *et al.* [2] reported a similar case of a penetrating ear trauma due to a tree branch that caused PLF with stapes fracture, and dislocation inside the vestibule. Treatment consisted of surgical removal of the fractured stapes from the vestibule, sealing the fistula with perichondrium and ossicular chain reconstruction with a tragal cartilage columella. Uehara *et al.* [2] also reported significant improvement in vestibular and cochlear function.

Despite differences in the surgical approach (surgical decision of stapes removal, choice of graft material for fistula repair, and ossicular chain reconstruction), most of the cases reported in the literature achieved control of vestibular symptoms after surgery. However, hearing outcomes were highly variable [1, 2, 5–9]. Prognostic factors for restoration of hearing function include: time interval to surgery, initial bone conduction thresholds, and presence of stapes fractures suggesting the size of the fistula and the amount of damage to the inner ear [6].

## Conclusion

Management of traumatic PLF with stapedovestibular dislocation remains controversial. Surgical intervention is indicated in case of persistent cochlear and or vestibular symptoms, to locate and seal the fistula and prevent further inner ear damage. Stapes condition is a key factor for surgical decision. Hearing outcomes are essentially conditioned by early diagnosis and management, and associated middle or inner ear injuries.
